# Dissection of action mechanisms of Zuogui Pill in the treatment of liver cancer based on machine learning and network pharmacology: A review

**DOI:** 10.1097/MD.0000000000035628

**Published:** 2023-10-20

**Authors:** Biao Guang, Xiang Gao, Xiangrong Chen, RuiLing Li, Li Ma

**Affiliations:** a College of Information Engineering, Hubei University of Chinese Medicine, Wuhan, China; b Institute of Liver Disease, Hospital of Hubei University of Chinese Medicine, Wuhan, China; c Affiliated Hospital of Hubei University of Chinese Medicine, Wuhan, China; d School of Foreign Language, Hubei University of Chinese Medicine, Wuhan, China.

**Keywords:** Chinese medicine, clustering analysis, liver cancer, network pharmacology, Zuogui Pill

## Abstract

This study aimed to investigate the underlying mechanism of Zuogui Pill in its efficacy against liver cancer, employing a combination of data mining approaches and network pharmacology methods. A novel clustering analysis algorithm was proposed to identify the core gene modules of Zuogui Pill. This algorithm successfully identified 5 core modules, with the first large module comprised of twelve proteins forming a 12-clique, representing the strongest connections among them. By utilizing GEO platform, ten key target proteins were detected, including FOS, PTGS2, and MYC. According to the GO annotation and KEGG analysis, desired target proteins were significantly enriched in various biological processes (BP). The analysis showed that ten key targets were strongly associated with signaling pathways mainly centered on MAPK and PI3K-Akt pathway. Additionally, molecular docking revealed strong binding affinities between core active ingredients of Zuogui Pill and these key targets, and the best affinity modes were observed for PTGS2-Sesamin, PRKCA-Sesamin, FOS-delta-Carotene. In order to establish the relationships between clinical symptoms and drug targets, a heterogeneous targets-related network was constructed. A total of 60 key target-symptom association pairs were detected, exemplified by the strongly association between fever and PTGS2 through the intermediary of Shu Di Huang. In summary, symptom-target associations are valuable in uncovering the underlying molecular mechanisms of Zuogui Pill. Our work reinforced the notion that Zuogui pill exhibits therapeutic potential on liver cancer through network targets, as well as synergistic effects of multi-component and multi-pathway. This study provided specific references for future experiments at the cost of less time.

## 1. Introduction

Liver cancer is one of the high-incidence malignant diseases within the digestive system, and approximately, 905,677 new cases of liver cancer were estimated worldwide in 2020 according to the estimation of the World Health Organization.^[1]^ The etiology is commonly attributed to be the synergistic effect of multiple oncogenes and gene mutation with multi-factor synergy, though the pathogenic mechanisms remain unclear. Surgical intervention is often employed for liver cancer in modern medicine. Unfortunately, the high reoccurrence rate and poor prognosis still pose challenging. Drawing on the principle of syndrome differentiation and treatment, Traditional Chinese Medicine (TCM) serves as a complementary therapy on liver cancer. By simultaneously regulating multiple components and multiple targets, TCM not only relieves severe postoperative symptoms but also endeavors to prevent the reoccurrence and improve prognosis.^[2]^ It has been known that Zuogui Pill has the certain research basis in treatment of liver cancer. Li et al^[3]^ proposed that it can effectively regulate liver cell proliferation and apoptosis, and mitigate liver tissue damage based on the experimental results. However, the mechanism underlying the therapeutic impact on liver cancer still cannot be clarified. The emergence of modular pharmacology in the biomedical network has introduced new avenues of research concerning the complex mechanism by which TCM exerts its therapeutic effects, employing multi-target and multi-pathway approaches.^[4,5]^ In this study, we constructed the protein-protein interaction (PPI) network via the common target genes of Zuogui Pill and liver cancer. Subsequently, potential functional modules were identified using clustering analysis algorithm. We aimed to uncover the key targets of Zuogui Pill in combating liver cancer and elucidate the molecular mechanism so that a valuable reference could be provided for future experimental studies.

## 2. Materials and methods

**Figure 1.**
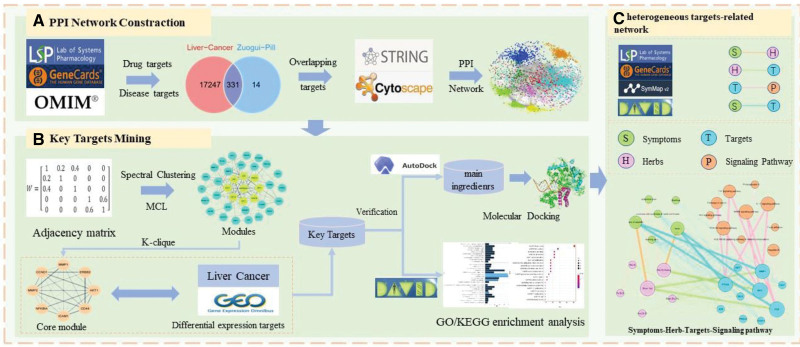
The workflow of this study based on machine learning and network pharmacology.

The workflow on the combination of machine learning and network pharmacology in this study was seen in Figure 1.

### 2.1. Screening of active ingredients and target proteins of Zuogui Pill

The active ingredients of Zuogui Pill were searched in the Traditional Chinese Medicine Systems Pharmacology Database and Analysis Platform (TCMSP, http://tcmspw.com/tcmsp.php),^[6]^ which aimed to analyze the biological mechanism of Chinese herbs at the genetic level.^[7]^ Zuogui Pill is mainly composed of 6 herbs including Shu Di Huang (Rehmanniae Radix Praeparata), Tu Si Zi (Cuscutae Semen), and Niu Xi (Achyranthis Bidentatae Radix). Firstly, the active ingredients of Zuogui Pill were screened if both oral bioavailability (OB ≥ 30%) and drug likeness (DL ≥ 0.18) were assumed. Secondly, the target proteins corresponding to ingredients were extracted in the TCMSP, and then, the selected targets were converted into the SwissProt identifiers in the UniProt database (https://www.uniprot.org/).

### 2.2. Obtaining the overlapping target genes between Zuogui Pill and liver cancer

The keywords “Liver Cancer” and “hepatocellular carcinoma” were searched in GeneCards platform (https://www.genecards.org/) and the Online Mendelian Inheritance Man platform (OMIM, https://omim.org/), genes related to liver cancer were obtained after removing the duplicate ones. The overlapping targets between liver cancer and Zuogui Pill in Venny2.1 platform (https://bioinfogp.cnb.csic.es/tools/venny/index.html) were identified as potential targets in the treatment of liver cancer.

### 2.3. Clustering analysis

The common genes of Zuogui Pill and liver cancer were uploaded into the Retrieval of Interacting Genes database (STRING, https://string-db.org/), and the data on the PPI was obtained. w_ij_ was defined as the interaction coefficient between protein node N_i_ and N_j_, d_i_ represented the sum of the weighted degree of node N_i_, it was calculated as follows:

$$\begin{array}{l} d_{i}=\overset{n}{\underset{j=1}{\sum }}W_{ij}\left(i=1,2,3,4\cdots n\right) \end{array}$$ (1)

To identify the functional modules in the PPI network, we proposed a 2-stage clustering algorithm based on Spectral clustering method and Markov Cluster (MCL) algorithm. The spectral clustering algorithm was conducted in the first phase, which has good property because of low sensitivity to the shape of the sample space and adaptability for sparse data.^[8]^ The adjacency matrix W is constructed with aforementioned *w*_*ij*_, namely as W = (*w*_*ij*_). The diagonal matrix D with *d*_*ii*_ equal to the sum of the elements of row i of W. The Laplace matrix L was defined as follows: L = D-W. And the matrix L was standardized as *D*^*−*1*/*2^*LD*^*−*1*/*2^, and the eigenvalues and corresponding eigenvectors of L was calculated. The eigenvectors corresponding to the k largest eigenvalues are stacked as columns to form the n × k matrix U. Each row of U is re-normalized to unit length to matrix Y. The matrix Y is a real valued solution of the clustering problem, the number of columns of Y, k, is defined as the number of clusters. Subsequently, we employed the MCL algorithm in the second phase, which is a graph clustering algorithm based on Markov Chain proposed by van Dongen.^[9]^ Initially, the adjacency matrix of the network is converted into a column stochastic matrix by dividing every non-zero entry by the sum of all entries in the column where the entry belongs. And then the network was divided into communities by alternately applying 2 operators named expansion and inflation on the matrix, until the matrix converges.

The proposed method integrates the advantages of spectral clustering and MCL algorithm, enabling efficient mining of functional modules from noisy data. To identify core modules, we applied the k-clique algorithm^[10]^ to search for the largest clique in each cluster. The clusters with less than 3 targets were discarded. Gephi 0.9.3 and Cytoscape 3.9.1 software were used to visualize the PPI network, Spectral clustering method and MCL algorithm were utilized via R studio software.

### 2.4. Differential expression analysis

The GEO database platform was used to retrieve the gene expression data of liver cancer. The species was set as “Homo Sapiens.” The differential expression analysis was performed by using the GEO2R platform, and differentially expressed genes (DEGs) with the cutoff criteria of |Log2FC|>1 and

*P* value < .05 and visualized by Rstudio software.

### 2.5. GO and KEGG enrichment analysis

Gene Ontology (GO) and Kyoto Encyclopaedia of Genes and Genomics (KEGG) enrichment analysis were used to validate the biological function of key targets. Functional analysis and pathway enrichment were performed on the key targets using DAVID database for annotation, visualization and integrated discovery (https://david.ncifcrf.gov/). The threshold value *P ≤ .*05 was considered statistically significant.

### 2.6. Molecular docking

Ten key targets and the top 5 active ingredients of Zuogui Pill were selected. The 3D structure of key target was downloaded from PDB database (https://www.rcsb.org). The target proteins were processed by removing water motifs and adding hydrogen by AutoDockTools-1.5.7. The protein receptors and ligand were converted to PDBQT format files. The lower the values of binding energy, the more stable the binding between proteins and active ingredients. It was generally believed that the binding energy less than −7.0 kcal/mol indicate stable binding affinity.

### 2.7. Collection of TCM symptoms and clinical phenotypes

TCM symptoms were collected from SymMap platform (http://www.symmap.org/),^[11]^ treated by both TCM and modern medicine related to 6 herbs of Zuogui Pill, including Shu Di Huang (Rehmanniae Radix Praeparata), Shan Zhu Yu (Corni Fructus), Niu Xi (Achyranthis Bidentatae Radix), Gou Qi Zi (Lycii Fructus), and Tu Si Zi (Cuscutae Semen). Clinical phenotypes related to liver cancer were collected using MalaCards and Orphanet databases.

## 3. Results

### 3.1. The candidate targets and active ingredients of Zuogui Pill on liver cancer

**Figure 2.**
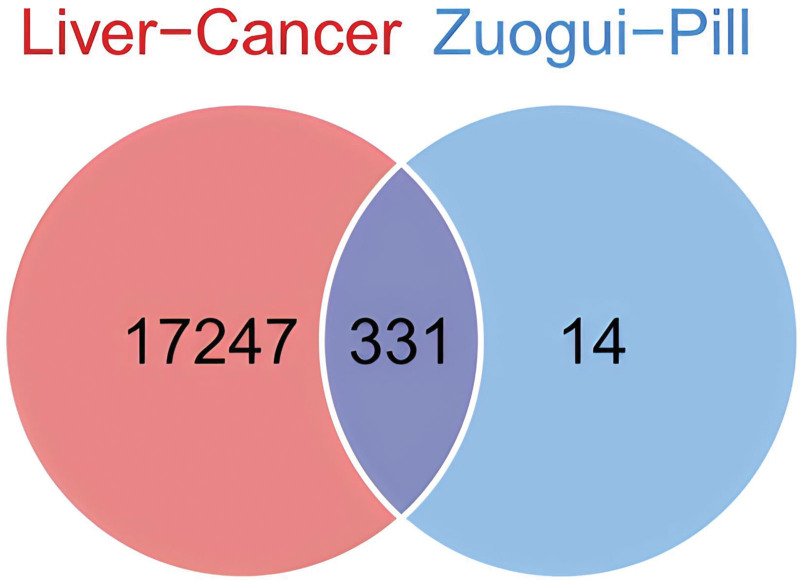
The Venn diagram of 331 overlapping targets between Zuogui Pill and liver cancer.

After deleting duplicate ingredients, a total of 65 active ingredients of Zuogui Pill were searched from TCMSP platform, 2 of which were found from Shu Di Huang (Rehmanniae Radix Praeparata), 45 from Gou Qi Zi (Lycii Fructus), 20 from Niu Xi (Achyranthis Bidentatae Radix), 16 from Shan Yao (Rhizoma Dioscoreae), 11 from Tu Si Zi (Cuscutae Semen). As Figure 2 showed, 17,578 target genes of liver cancer were selected, 345 target proteins of Zuogui Pill were screened. And then, 331 overlapping targets between Zuogui Pill and liver cancer were identified.

### 3.2. Core modules of Zuogui Pill in treating liver cancer

331 shared target genes of Zuogui Pill and liver cancer were used to construct PPI network, which was composed of 331 nodes and 5613 edges. The thickness of the edge indicated the strength of interaction between 2 nodes. Three topological features were calculated to show the overall centrality of PPI network, the mean clustering coefficient was 0.526, the density was 0.103, and the network centrality (NC) was 0.387. Besides, the weighted degree of each node was calculated, the maximum and minimum were 115.18 and 1.42 respectively, and the mean degree was 22.61.

**Figure 3.**
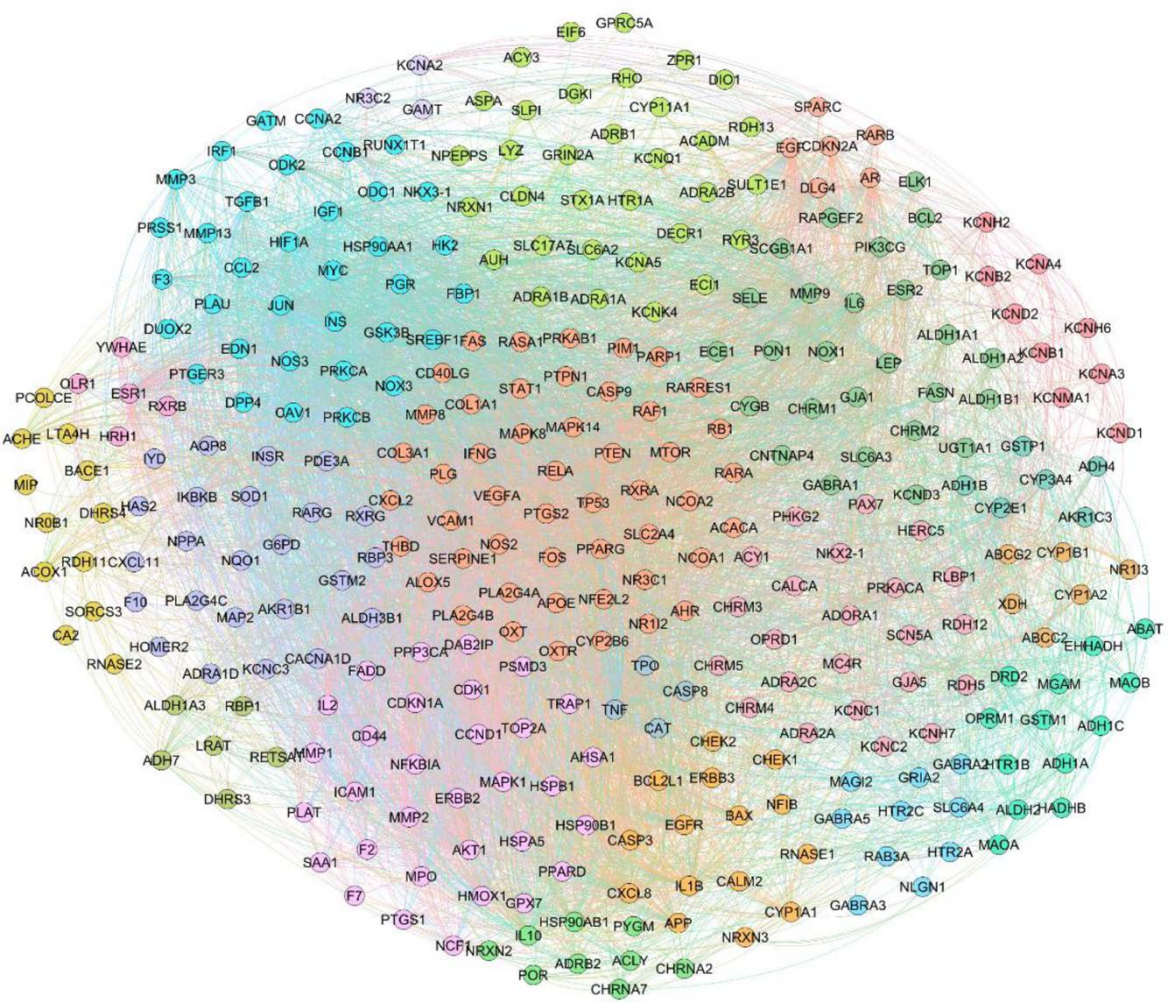
The result of clustering analysis based on 331 target proteins. A total of 20 clusters were obtained through our proposed clustering analysis, and different colors represented different clusters.

**Figure 4.**
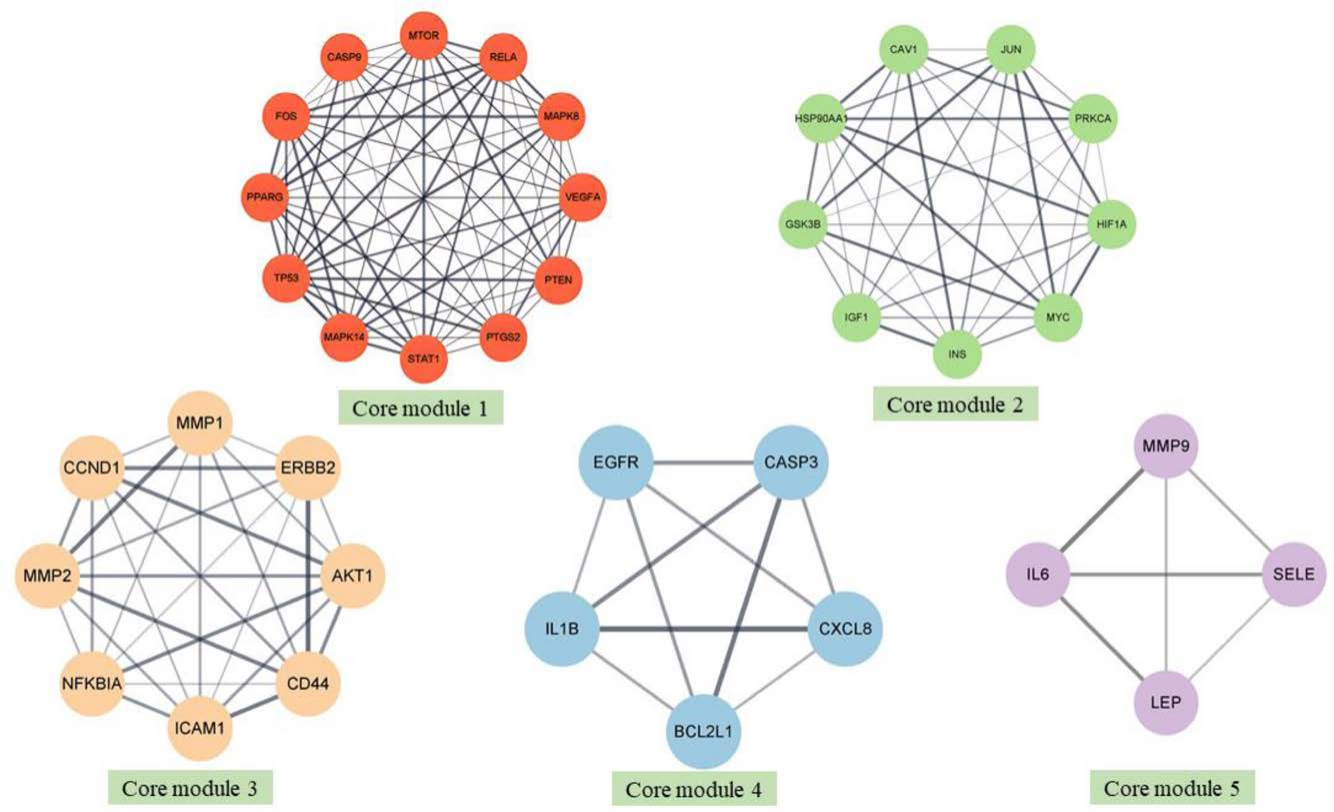
Five core modules of Zuogui Pill in the treatment of liver cancer.

Based on the novel 2-stage clustering analysis, the original PPI network was divided into 20 communities, as it was shown in Figure 3. According to the ranking number of k-cliques (k > 3), 5 cliques were identified as core modules, as they were shown in Figure 4. For example, the core module 1was comprised of a 12-clique containing twelve targets, such as PTGS2, FOS, TP53 et al. Similarly, the core module 2 was composed of 9 protein targets, including IGF1, JUN, MYC, CAV1 et al, the module 3 containing 8 protein targets, mainly including AKT1, MMP1. Another 2 core modules were also shown in Figure 4.

### 3.3. identification of key target proteins of Zuogui Pill in treating liver cancer

**Figure 5.**
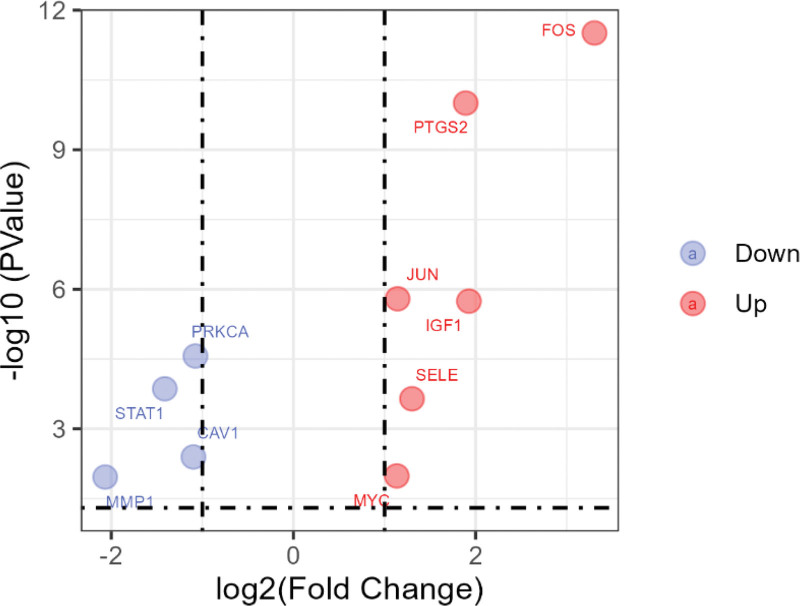
Volcano plot of ten key targets. Six red targets were upregulated genes, 4 blue targets were downregulated genes. DEGs were defined with the cutoff criteria of |Log2FC|> 1 and *P* value < .05. DEGs = differentially expressed genes.

Three liver cancer-related gene expression datasets GSE60502, GSE84402, and GSE112790 were retrieved through the GEO platform. A total of 1870 differentially expressed genes (DEGs) were obtained from the GSE60502 dataset, 969 of which were up-regulated and 811 were down-regulated. Similarly, 1566 of DEGs were obtained from the GSE84402 dataset, 2361 from the GSE112790 dataset. Subsequently, 2986 of DEGs were included after all of them were merged. 38 target proteins contained in 5 core modules were mapped to 2986 DEGs. And then, 10 proteins were identified as key targets of Zuogui Pill against liver cancer, including FOS, PTGS2, STAT1, IGF1, MYC, JUN, PRKCA, CAV1, MMP1, and SELE. Furthermore, 4 targets were marked as down-regulated, another 6 ones were up-regulated. They were seen in Figure 5.

### 3.4. GO and KEGG enrichment analysis of key targets

**Figure 6.**
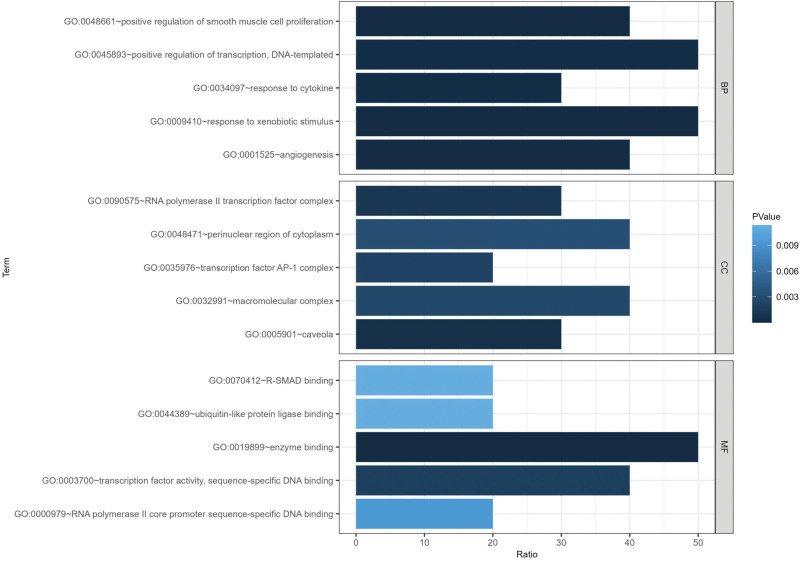
GO enrichment analysis of 10 key targets. The horizontal axis represents the ratio, the vertical axis represents the biological functions, and the color represents the significance. The darker the color, the more significant. GO = Gene Ontology.

According to the GO annotation analysis, a total of 71 GO enrichment items were significantly obtained (*P* ≤ .05). 53 of these items were involved in biological process (BP), such as positive regulation of smooth muscle cell proliferation (GO:0048661), response to xenobiotic stimulus (GO:0009410), angiogenesis (GO:0001525), positive regulation of transcription, DNA-templated (GO:0045893), and response to cytokine (GO:0034097). Seven cell component (CC) items, were mainly related to caveola (GO:0005901), RNA polymerase II transcription factor complex (GO:0090575), and transcription factor AP-1 complex (GO:0035976). Additionally, 11 molecular function (MF) entries were identified, mainly including enzyme binding (GO:0019899), transcription factor activity, sequence-specific DNA binding (GO:0003700), and RNA polymerase II core promoter sequence-specific DNA binding (GO:0000979). These results showed that key targets were involved in different biological functions. According to the *P* value, the top 5 GO entries in each category were shown in Figure 6.

**Figure 7.**
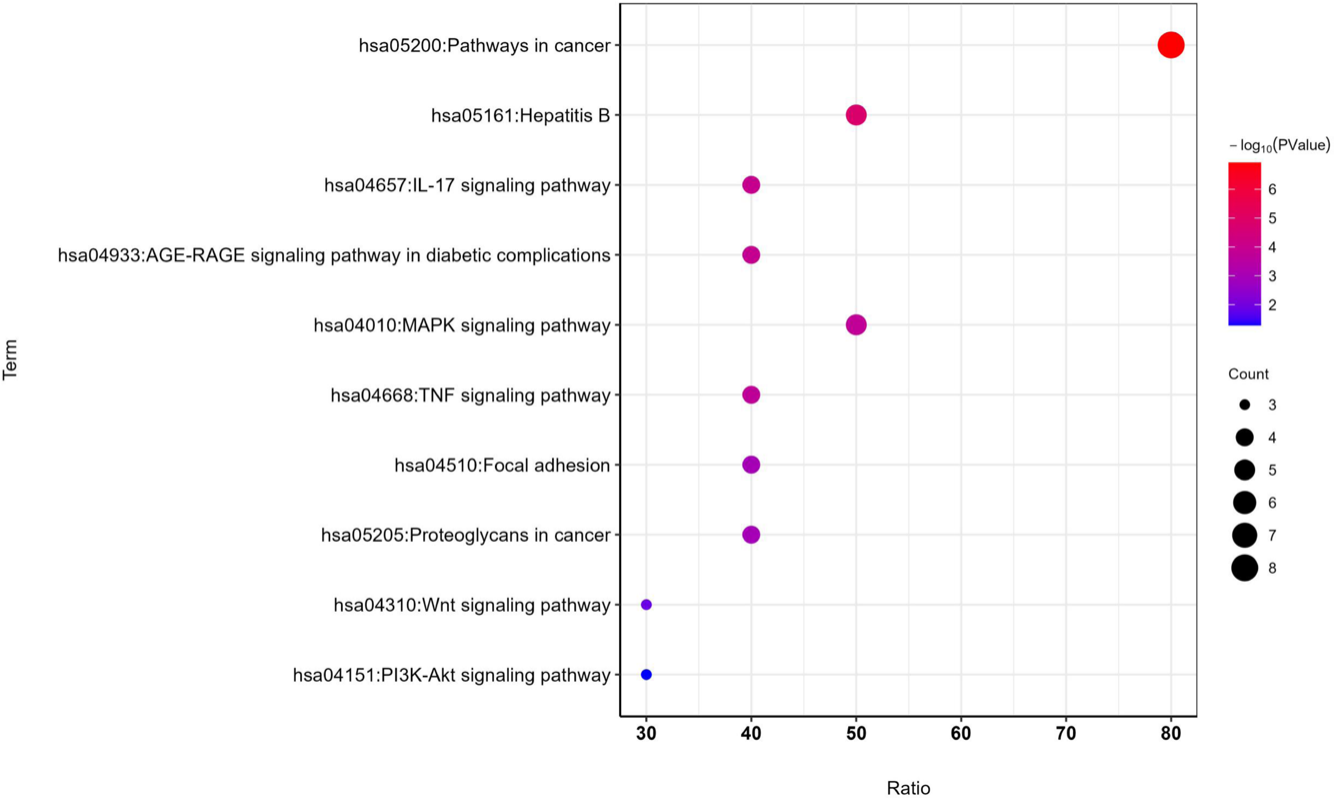
KEGG enrichment analysis of 10 key targets. The horizontal axis represents the ratio, the vertical axis represents the signaling pathway, and the color represents the significance.

Furthermore, a total of 43 KEGG enrichment entries were significantly obtained, considering the signaling pathways in ascending order of the *P* value. The correlation between the top 10 signaling pathways and 10 key targets were shown in Table 1 and Figure 7. Many signaling pathways, such as Hepatitis B, MAPK, IL-17 signaling pathways, were significantly associated with most of key targets. Additionally, PRKCA, JUN, MYC were enriched in most of the top 10 signaling pathways, closely related to the proliferation and apoptosis of liver cell.

**Table 1:** Correlation between top 10 KEGG pathways and 10 key targets.

KEGG pathways	FOS	PTGS2	STAT1	IGF1	MYC	JUN	PRKCA	CAV1	MMP1	SELE	Sum
hsa05200:Pathways in cancer	1	1	1	1	1	1	1	0	1	0	8
hsa05161:Hepatitis B	1	0	1	0	1	1	1	0	0	0	5
hsa04010:MAPK signaling pathway	1	0	0	1	1	1	1	0	0	0	5
hsa04657:IL-17 signaling pathway	1	1	0	0	0	1	0	0	1	0	4
hsa04668:TNF signaling pathway	1	1	0	0	0	1	0	0	0	1	4
hsa04933:AGE-RAGE signaling pathway in diabetic complications	0	0	1	0	0	1	1	0	0	1	4
hsa04510:Focal adhesion	0	0	0	1	0	1	1	1	0	0	4
hsa05205:Proteoglycans in cancer	0	0	0	1	1	0	1	1	0	0	4
hsa04310:Wnt signaling pathway	0	0	0	0	1	1	1	0	0	0	3
hsa04151:PI3K-Akt signaling pathway	0	0	0	1	1	0	1	0	0	0	3
Sum	5	3	3	5	6	8	8	2	2	2	

The value is 1 if the target is significantly related to the signaling pathway, otherwise, the value is 0.

KEGG = Kyoto Encyclopaedia of Genes and Genomics.

### 3.5. Verification of 10 key targets through molecular docking

The protein structures of ten hub targets were acquired from PDB database, including FOS (PDB ID: 6s1t), PTGS2(PDB ID: 3nt1), STAT1(PDB ID: 3su6), IGF1(PDB ID: 1wqj), MYC (PDB ID: 7r2h), JUN (PDB ID: 2p33), PRKCA (PDB ID: 4ra4), CAV1(PDB ID: 7luc), MMP1(PDB ID: 3shi) and SELE (PDB ID: 5jha). In addition, the top 5 active ingredients were selected, including quercetin (MOL000098), beta-sitosterol (MOL000359), delta-Carotene (MOL010234), sesamin (MOL001558) and matrine (MOL005944). It was displayed from Table 2 that the best 3 of affinity modes were PTGS2-sesamin (−9.7 kcal/mol), FOS-quercetin (−9.7 kcal/mol) and PRKCA-sesamin (−9.6 kcal/mol), and there were 8 key targets with the average binding energy less than −7.0 kcal/mol. Detailed results were shown in Table 2.

**Table 2:** The binding energy between active ingredient and key target.

Active ingredient	Binding energy (kcal/mol)									
	FOS	PTGS2	STAT1	IGF1	MYC	JUN	PRKCA	CAV1	MMP1	SELE
Quercetin	−9.7	−9.2	−6.8	−8.7	−6.5	−7.2	−7.5	−8.0	−8.8	−8.4
Beta-sitosterol	−8.6	−9.2	−5.4	−6.8	−6.1	−8.0	−7.0	−8.6	−7.5	−8.3
Delta-Carotene	−9.2	−8.9	−5.5	−7.3	−5.5	−6.9	−6.2	−7.7	−6.7	−6.9
Sesamin	−8.8	−9.7	−6.7	−8.7	−7.9	−8.5	−9.6	−9.2	−9.1	−9.2
Matrine	−8.3	−8.5	−5.3	−6.5	−5.5	−6.3	−6.6	−7.8	−6.6	−6.9
Mean	−8.9	−9.1	−5.9	−7.6	−5.6	−7.4	−7.4	−8.3	−7.7	−7.9

### 3.6. Construction of symptom-herb-target-signaling pathway association network

**Figure 8.**
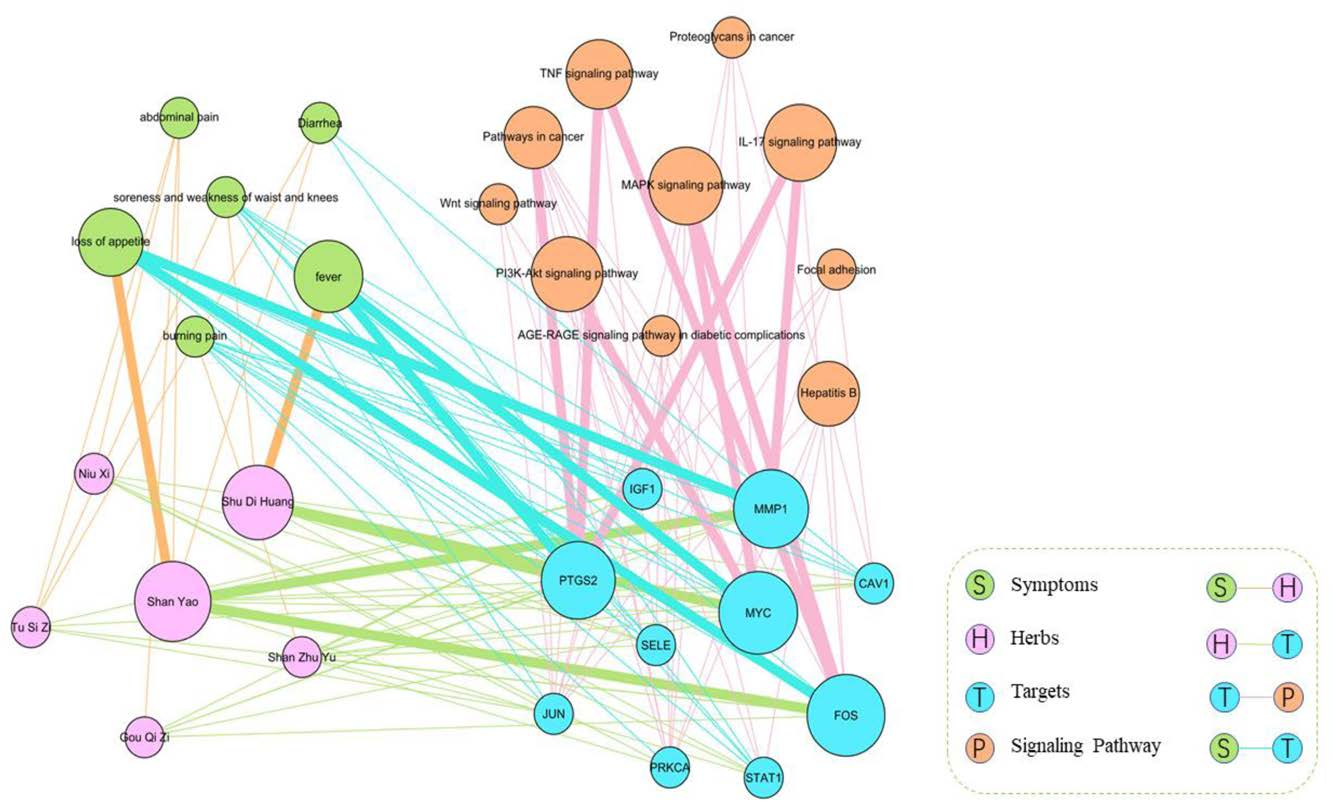
The symptom-herb-target-signaling pathway association network. Different colors represented different types of nodes and different connections.

To explore the underlying relationship between clinical symptoms and medication use, we mapped 34 Chinese terms of clinical symptoms corresponding to Zuogui Pill into 56 English terms of phenotypes related to liver cancer. And then, 6 clinical symptoms were successfully mapped, including fever, abdominal pain, loss of appetite, soreness and weakness of waist and knees, burning pain, diarrhea. According to the herb-symptom association network,11 herb-symptom association pairs were detected, such as Shu Di Huang to fever, Shan Yao to loss of appetite, and Tu Si Zi to soreness and weakness of waist and knees. Furthermore, based on herb-target association pairs, the relationship network was built between clinical symptoms and key targets. Subsequently, 60 clinical symptom-target pairs were identified. To elucidate the indirect relationships between TCM symptoms and targets, a heterogeneous key targets-related network was seen in Figure 8. We found that each key target was associated with different symptoms. For example, PTGS2 revealed the highest diversity with 5 associated symptoms. On the other hand, each clinical symptom was associated with more than 1 key target. Additionally, MAPK, IL-17 and PI3K-Akt signaling pathways were associated with different key targets, according to the target-signaling pathway association network.

## 4. Discussion

Zuogui Pill originated from the work “Jingyue Quanshu” authored by Zhang Zhongjing, a renowned clinician during the Ming Dynasty, and has been widely employed in clinical practice due to its outstanding effect, specifically, to improve clinical symptoms on liver cancer. In recent years, many studies^[12–14]^ have delved into the molecular mechanism of Zuogui Pill in treating Osteoporosis and Ovarian Dysfunction, and Gao et al^[15]^ further substantiated the multi-target and multi-level synergistic effects of active ingredients in the Zuogui Pill, employing a system biology approach. As we know, the holistic perspective of TCM intrinsically aligns with the systematic viewpoint of bioinformatics. Consequently, the utilization of computational algorithms for the identification of key protein targets and the determination of biological pathways in Chinese formulas has emerged as a prominent research area.^[16]^ In our study, we proposed a novel 2-phase community-identification algorithm that integrated spectral clustering and MCL algorithm. These 2 algorithms have been applying to the identification of modules within real PPI network.^[17,18]^ As anticipated, ten key targets identified by 5 core modules encompass a substantial portion of biological function associated with Zuogui Pill, combining topological properties of PPI network. These key targets well expressed the essentiality feature for protein-protein interaction, because they were all included in the largest k-clique of each module in PPI network. In addition, these key protein targets revealed different biological functions by using GEO database. Among them, PTGS2 can activate PINK1/Parkin-mediated mitophagy and accelerate apoptosis of Hepatoma cell.^[19]^ FOS, MYC and JUN have been testified to regulate the proliferation and apoptosis of hepatoma cells in present experimental results.^[20]^ Besides, FOS modulated plasticity of epithelial hepatocytes and acted tumor suppressive in neoplastic hepatocytes by stimulating cell cycle inhibition and cell death,^[21]^ IGF1 could inhibit the malignant progression of HCC.^[22]^ It was reported that Hepatitis B virus X protein (HBx) induces hepatocarcinogenesis via PKCα-mediated overexpression of cytoplasmic p21.^[23]^ Furthermore, STAT1 exerts tumor-suppressive effected in hepatocarcinogenesis through induction of G0/G1 cell cycle arrest and apoptosis.^[24]^ These key targets validated the multi-target molecular mechanism for Zuogui Pill in the treatment of liver cancer.

According to the GO and KEGG enrichment analysis, it was found that key targets played different roles in expressing biological functions. Over 50% of key targets, such as PTGS2, FOS, were involved in Hepatitis B, MAPK signaling pathways, which were closely associated with liver cancer. Furthermore, PRKCA, JUN enriched in most of TOP 10 KEGG pathways, such as IL-17, MAPK, PI3K-Akt signaling pathways. PI3K-Akt and MAPK pathways make a significant impact on regulating the cell cycle, proliferation, apoptosis, and metabolism.^[25–27]^ On the other hand, key protein targets tended to have interactions among different key modules. For example, PTGS2, JUN, SELE were all involved in TNF signaling pathway. LPS can trigger the release of inflammatory mediators like PTGS2.^[28]^ Zuo et al^[29]^ demonstrated that Cr (VI) induced PTGS2 expression via an NFκB/c-Jun/AP-1-dependent pathway. Therefore, these key targets have significant correlations within important cancer-related cellular processes. In addition, the analysis of molecular docking showed that the top 5 active ingredients of Zuogui Pill commonly had good binding energy with 10 key targets, results based on combining machine learning and network pharmacology were further verified for detecting key targets. It was suggested that sesamin and quercetin may give full play to the treatment of liver cancer.

In real-world TCM clinical setting, symptoms provide key information for personalized herb treatment.^[30]^ Therefore, symptom-gene associations may help to uncover the molecular mechanism for Chinese formula. To elucidate the potential mechanism of Zuogui Pill in treating liver cancer from the perspective of improving symptoms, we mapped the associated symptoms of Zuogui Pill to phenotype terms of liver cancer. It was found that the clinical symptoms treated by Zuogui Pill are similar to the phenotypes of liver cancer. Based on the symptom-gene-herb-signaling pathway associations, particularly, PTGS2 exhibit correlations with fever, the clinical manifestation of liver cancer, thereby suggesting that the administration of Shu Di Huang, a key constituent of Zuogui Pill, may effectively mitigate fever by modulating PTGS2 on IL-17 signaling pathway. Likewise, it was displayed that Shan Yao may improve loss of appetite by regulating FOS via MAPK signaling pathway. Zuogui Pill can improve different clinical symptoms in treating liver cancer, by regulating multiply targets like PTGS2 and FOS, participating in MAPK, IL-17 and TNF signaling pathways.

Nevertheless, some limitations were inevitable in our study. 14 of 345 potential protein targets from ingredients of Zuogui Pill have been omitted, because they had not been found in the present databases related to liver cancer. It was necessary to be included into the further validation for exploring the complicated molecular mechanism of Zuogui Pill, maybe it would help to promote clinical applications in discovering new biological compound.

## 5. Conclusion

In summary, based on bioinformatics methods, we identified key drug targets of Zuogui Pill and revealed herb-target-symptom network correlations to explore the underlying therapeutic mechanism in the treatment of liver cancer. The results confirmed Zuogui Pill can mitigate clinical symptoms on liver cancer via synthetic mechanism of multi-component, multi-target and multi-pathway, our finding would provide promising evidence for further experimental verification of these hub targets.

## Author contributions

**Data curation:** Ruiling Li.

**Methodology:** Xiang Gao, Li Ma.

**Software:** Biao Guang.

**Visualization:** Biao Guang, Ruiling Li.

**Writing – original draft:** Biao Guang, Xiangrong Chen, Li Ma.

**Writing – review & editing:** Li Ma.
